# Rerefinement of the crystal structure of BiF_5_

**DOI:** 10.1107/S2056989024005759

**Published:** 2024-07-09

**Authors:** Tobias Burghardt Wassermann, Florian Kraus

**Affiliations:** a Philipps-Universität Marburg, Fachbereich Chemie, Hans-Meerwein-Str. 4, 35032 Marburg, Germany; Vienna University of Technology, Austria

**Keywords:** crystal structure, redetermination, bis­muth(V) fluoride

## Abstract

The crystal structure of bis­muth penta­fluoride, BiF_5_, was rerefined from single-crystal data. BiF_5_ crystallizes in the α-UF_5_ structure type in the form of colorless needles. In comparison with the previously reported crystal-structure model [Hebecker (1971 ▸). *Z. Anorg. Allg. Chem.***384**, 111–114], the lattice parameters and fractional atomic coordinates were determined to much higher precision and all atoms were refined anisotropically, leading to a significantly improved structure model. The Bi atom (site symmetry 4/*m*..) is surrounded by six F atoms in a distorted octa­hedral coordination environment. The [BiF_6_] octa­hedra are corner-linked to form infinite straight chains extending parallel to [001]. Density functional theory (DFT) calculations at the PBE0/TZVP level of theory were performed on the crystal structure of BiF_5_ to calculate its IR and Raman spectra. These are compared with experimental data.

## Abstract 2

Redetermination of the crystal structure of BiF_5_ was undertaken to a much higher precision and quantum chemical calculations for an assignment of the Raman and IR bands.

## 1. Chemical context

Bismuth(V) fluoride was first synthesized in the year 1940 (von Wartenberg *et al.*, 1940 ▸). Hebecker first determined its crystal structure in 1971 (Hebecker, 1971 ▸). During our studies of the chemistry of BiF_5_, we rerefined its crystal structure on basis of single-crystal data and recorded its IR and Raman spectra.

## 2. Structural commentary

**Figure 1:**
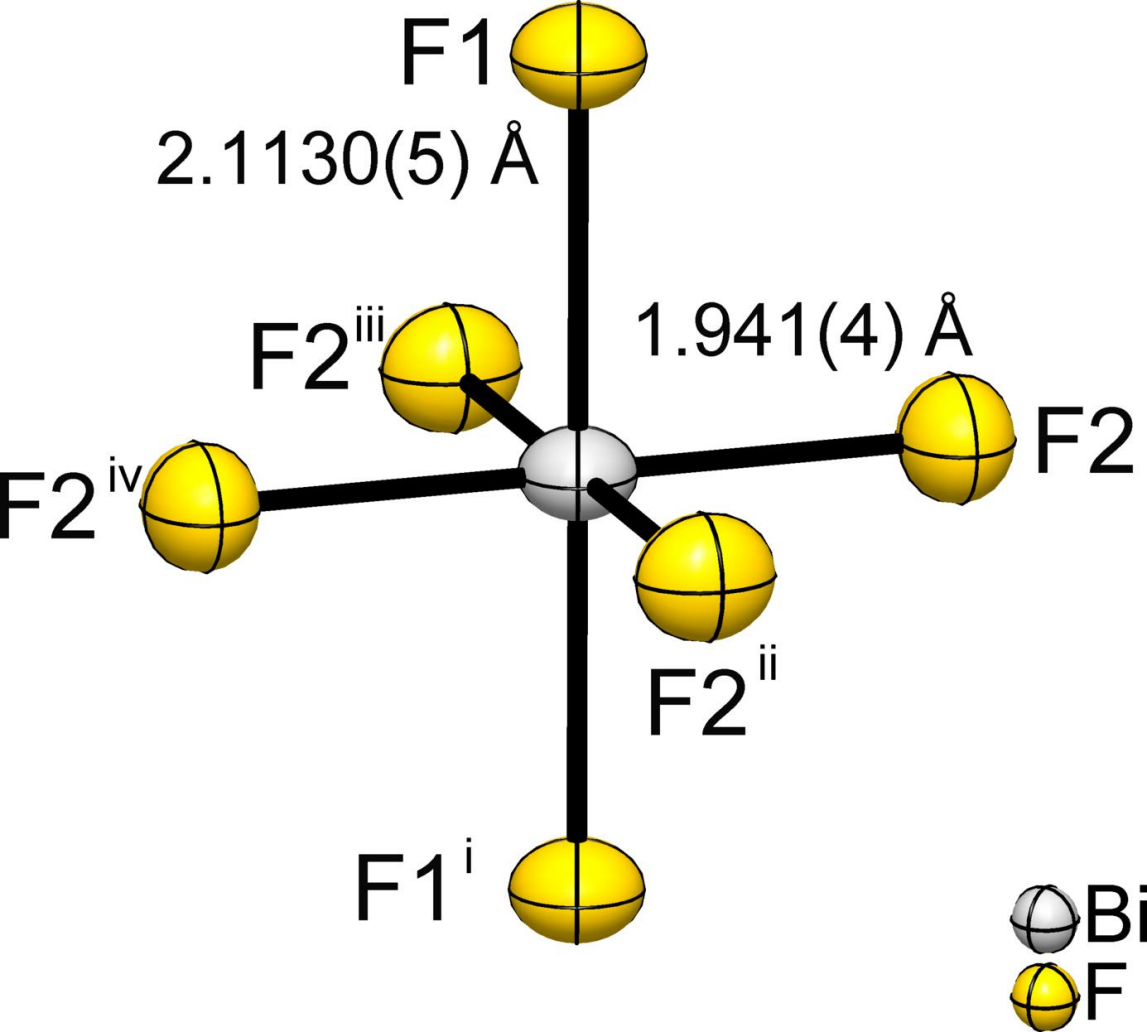
The coordination sphere of the Bi atom in the crystal structure of BiF_5_. Displacement ellipsoids are drawn at the 70% probability level. [Symmetry codes: (i) *x*, *y*, *z*  − 1; (ii) *y*, −*x*, *z*; (iii) −*y*, *x*, *z*; (iv) −*x*, −*y*, *z*.]

**Figure 2:**
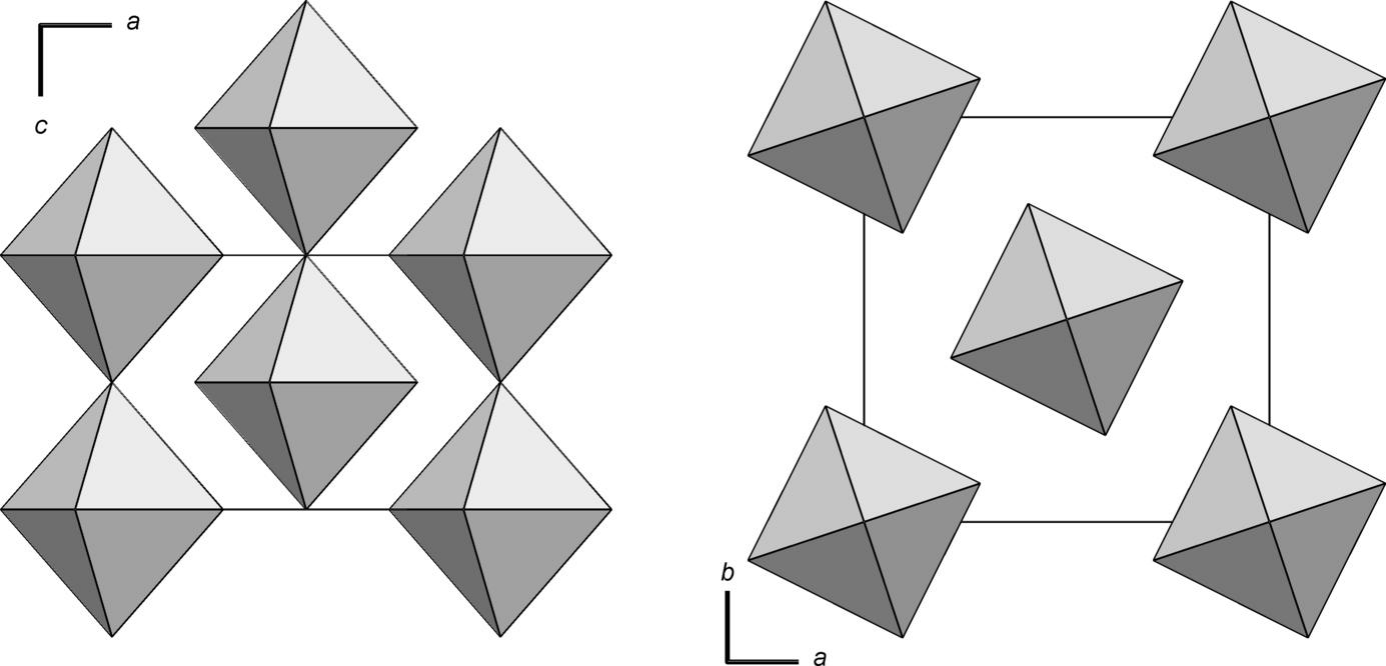
Crystal structure of BiF_5_ viewed along the *b* axis (left) and the *c* axis (right). The [BiF_6_] octa­hedra are shown in grey.

Bismuth(V) fluoride crystallizes in the tetra­gonal space group *I*4/*m*, Pearson symbol *tI*12 and Wyckoff sequence 87.*hba*. BiF_5_ adopts the *α*-UF_5_ structure type and exhibits Bi—F bond lengths of 1.941 (4) (4×) and 2.1130 (5) Å (2×). The structure consists of chains of *trans*-corner-sharing [BiF_6_] octa­hedra (point group symmetry 4/*m*..; Fig. 1 ▸) running parallel to the *c* axis. The chains can be described by the Niggli formula ^1^_∞_[BiF_4/1_F_2/2_]. The F1 atom bridges adjacent Bi atoms in the straight chain. As expected, the axial Bi—F1 bond lengths are longer than the four equatorial Bi—F2 bond lengths to the terminal ligands. Sections of the crystal structure showing the chains are shown in Fig. 2 ▸.

van der Waals contacts exist between neighbouring chains with inter­atomic distances of 2.944 (5) Å (F2⋯F2^v^) and 2.952 (5) Å (F1⋯F2^v^) [symmetry code: (v) 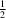 − *x*, 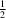 − *y*, 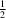 + *z*). Compared with the distances reported previously (Hebecker, 1971 ▸), these are 0.12 Å shorter for *d*(F2⋯F2) and 0.05 Å longer for *d*(F1⋯F2).

Regarding the crystal packing, a Bi atom is surrounded by 14 other Bi atoms in the shape of a distorted rhombic dodeca­hedron, which would correspond to the arrangement of the atoms in the W structure type. However, the rhombic dodeca­hedron is compressed due to the shorter intra­chain and the longer inter­chain Bi⋯Bi distances. The F1 atoms reside in the octa­hedral voids of the (idealized) body-centered cubic packing, while the F2 atoms are strongly displaced from these as the octa­hedra are rotated around the *c* axis (Fig. 2 ▸).

## 3. Vibrational spectra

**Figure 3:**
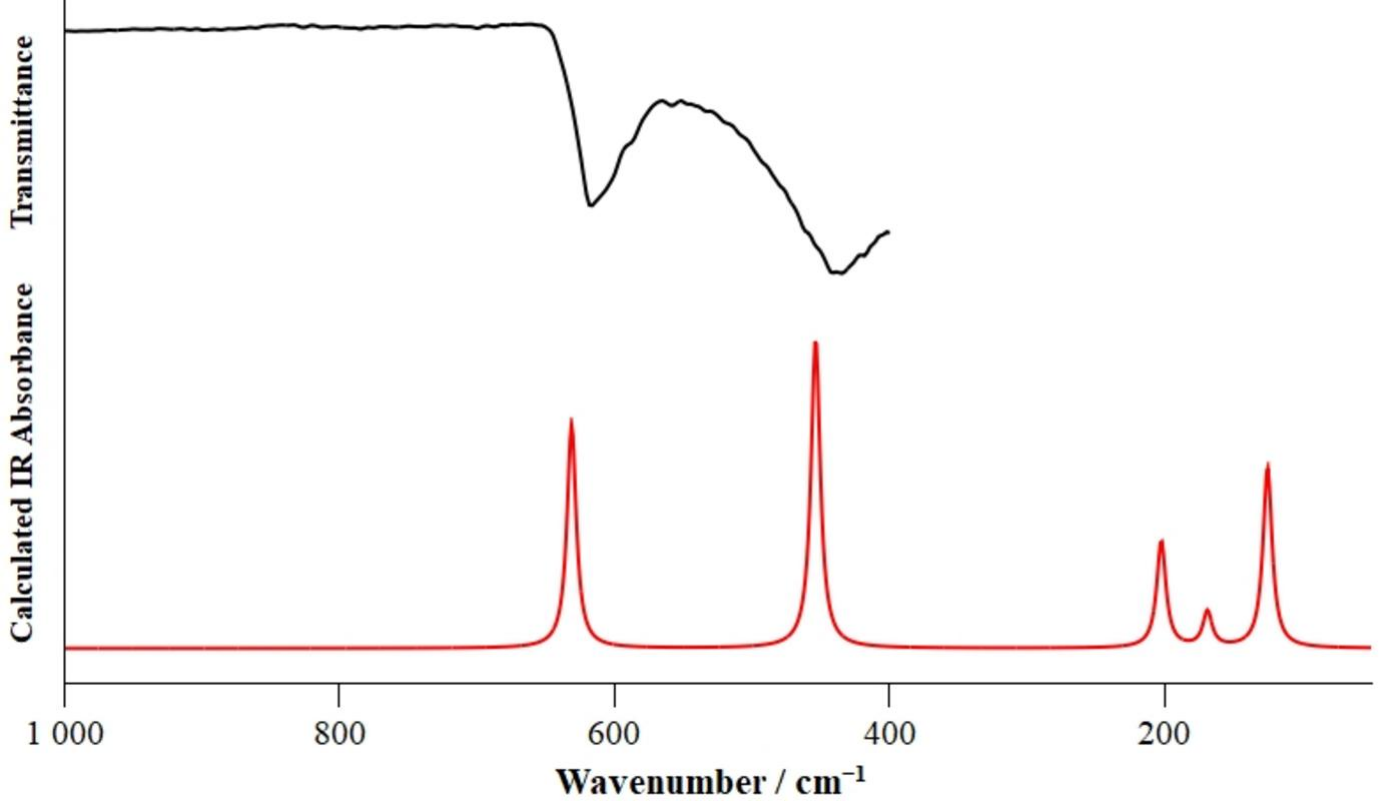
Recorded IR spectrum of crystalline BiF_5_ in black and calculated IR absorbance spectrum of solid BiF_5_ at the DFT-PBE0/TZVP level of theory in red. No bands were observed in the region from 1000 to 4000 cm^−1^.

IR and Raman spectra were recorded on a polycrystalline sample of BiF_5_ at 293 K. Quantum-chemical calculations at the DFT-PBE0/TZVP level of theory (Dovesi *et al.*, 2018 ▸; Zicovich-Wilson *et al.*, 2004 ▸; Pascale *et al.*, 2004 ▸; Maschio *et al.*, 2013 ▸) were performed on basis of the crystal structure of BiF_5_ to obtain the theoretical IR and Raman spectra. The recorded and calculated spectra are in good agreement, as shown in Figs. 3 ▸ and 4 ▸. A comparison of the observed and calculated bands is given in Table 1 ▸.

## 4. Synthesis and crystallization

Bismuth(III) fluoride (1.05 g, 3.95 mmol) was loaded in a corundum boat and placed inside a tube furnace. By passing diluted fluorine (F_2_/Ar, 10:90 *v*/*v*, Solvay) over the submitted material, bis­muth(V) fluoride was synthesized at 723 K, using a heating rate of 4 K min^−1^ from room temperature to 723 K and a holding time of 10 h while diluted fluorine was passed with a flow rate of 5 standard cubic centimeters per minute. After cooling down to room temperature, colorless needles of bis­muth(V) fluoride (601 mg, 1.98 mmol, 57%) were isolated.

## 5. Refinement

Crystal data, data collection and structure refinement details are summarized in Table 2 ▸.

## Supplementary Material

Crystal structure: contains datablock(s) I. DOI: 10.1107/S2056989024005759/wm5722sup1.cif

Structure factors: contains datablock(s) I. DOI: 10.1107/S2056989024005759/wm5722Isup2.hkl

CCDC reference: 2362790

Additional supporting information:  crystallographic information; 3D view; checkCIF report

## Figures and Tables

**Figure 4:**
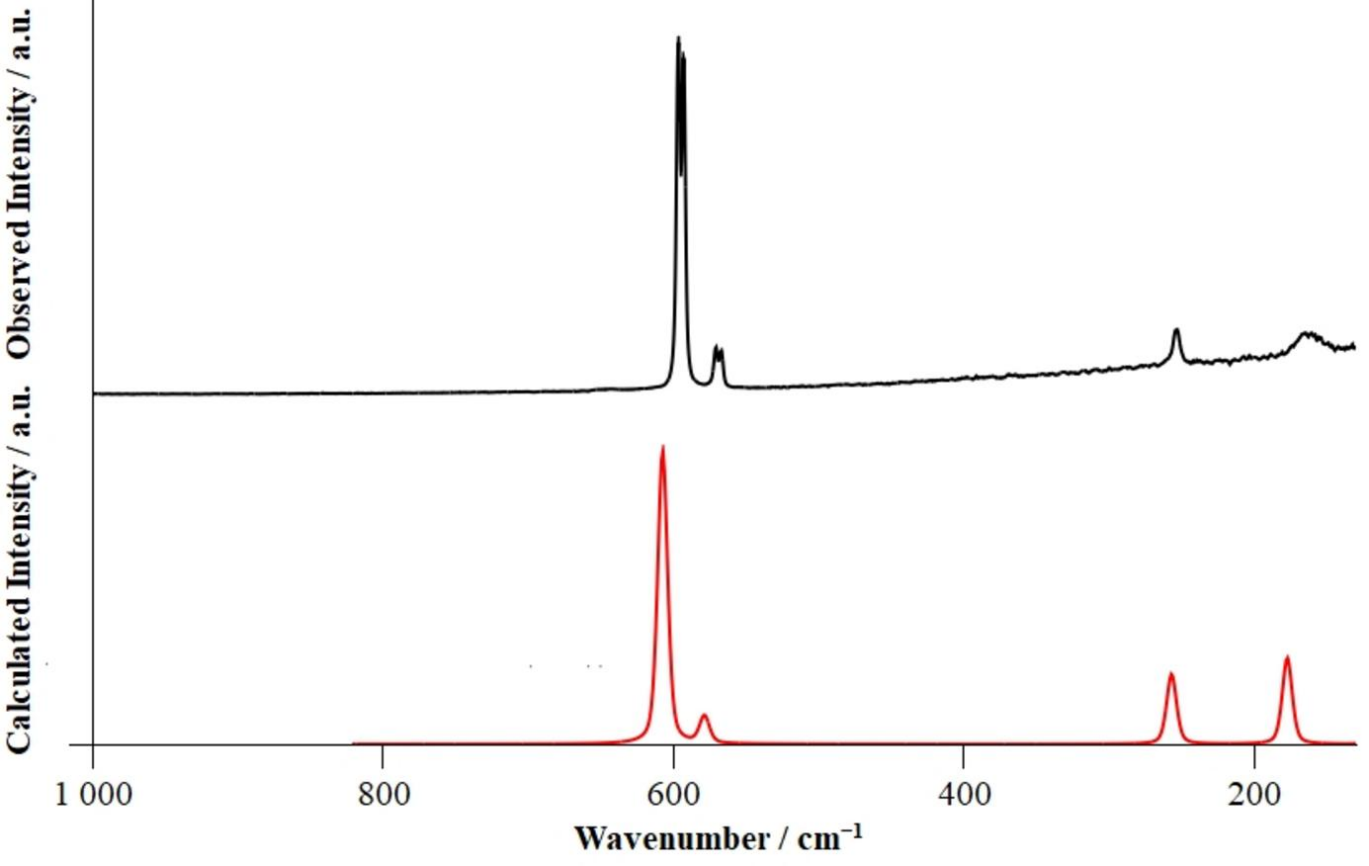
Recorded Raman spectrum (532 nm laser) of crystalline BiF_5_ in black, calculated Raman spectrum of solid BiF_5_ at the DFT-PBE0/TZVP level of theory in red. No bands were observed in the region from 1000 to 4000 cm^−1^.

**Table 1:** Band positions (cm^−1^) and band assignment of the IR and Raman spectra of solid BiF_5_ based on the calculated spectrum *ν* = stretching vibration, *δ* = bending vibration, *s* = symmetric, *as* = asymmetric, – = not observed.

IR		Raman		Assignment
*ν_calc_*	*v_obs_*	*ν_calc_*	*v_obs_*	
630	618	–	–	*v_as_*(Bi—F2) + *δ*(Bi—F1—Bi)
–	–	607	596, 593	*v_s_*(Bi—F2)
–	–	578	571, 567	*v*_a*s*_(Bi—F2)
453	441	–	–	*v_as_*(Bi—F_μ_)
–	–	256	254	*δ*(F2—Bi—F2)
202	–	–	–	*δ*(F—Bi—F)
168	–	–	–	*δ*(F—Bi—F)
–	–	177	164	*δ*(F2—Bi—F1)
125	–	–	–	*δ*(F2—Bi—F1) + *v_as_*(Bi—F1)

**Table 2:** Experimental details

Crystal data	
Chemical formula	BiF_5_
*M*_r_	303.98
Crystal system, space group	Tetragonal, *I*4/*m*
Temperature (K)	100
*a*, *c* (Å)	6.4439 (9), 4.2260 (9)
*V* (Å^3^)	175.48 (6)
*Z*	2
Radiation type	Mo *K*α
μ (mm^−1^)	49.84
Crystal size (mm)	0.31 × 0.13 × 0.11

Data collection	
Diffractometer	Stoe IPDS 2T
Absorption correction	Multi-scan (*LANA*; Koziskova *et al.*, 2016 ▸)
*T*_min_, *T*_max_	0.006, 0.078
No. of measured, independent and observed [*I* > 2σ(*I*)] reflections	1134, 174, 174
*R*_int_	0.027
(sin θ/λ)_max_ (Å^−1^)	0.742

Refinement	
*R*[*F*^2^ > 2σ(*F*^2^)], *wR*(*F*^2^), *S*	0.016, 0.042, 1.12
No. of reflections	174
No. of parameters	12
Δρ_max_, Δρ_min_ (e Å^−3^)	0.90, −1.38

Computer programs: *X-AREA* (Stoe, 2020 ▸), *SHELXT* (Sheldrick, 2015*a* ▸), *SHELXL* (Sheldrick, 2015*b* ▸), *DIAMOND* (Brandenburg & Putz, 2022 ▸), *publCIF* (Westrip, 2010 ▸) and *ShelXle* (Hübschle *et al.*, 2011 ▸).

## supplementary crystallographic information

### Bismuth pentafluoride Crystal data

**Table:**

BiF_5_	*D*_x_ = 5.753 Mg m^−^^3^
*M**_r_* = 303.98	Mo *K*α radiation, λ = 0.71073 Å
Tetragonal, *I*4/*m*	Cell parameters from 5333 reflections
*a* = 6.4439 (9) Å	θ = 8.8–60.0°
*c* = 4.2260 (9) Å	µ = 49.84 mm^−^^1^
*V* = 175.48 (6) Å^3^	*T* = 100 K
*Z* = 2	Needle, colorless
*F*(000) = 256	0.31 × 0.13 × 0.11 mm

### Bismuth pentafluoride Data collection

**Table:**

Stoe IPDS 2T diffractometer	174 independent reflections
Radiation source: sealed X-ray tube, 12 x 0.4 mm long-fine focus	174 reflections with *I* > 2σ(*I*)
Detector resolution: 6.67 pixels mm^-1^	*R*_int_ = 0.027
rotation method, ω scans	θ_max_ = 31.8°, θ_min_ = 4.5°
Absorption correction: multi-scan (*LANA*; Koziskova *et al.*, 2016)	*h* = −9→9
*T*_min_ = 0.006, *T*_max_ = 0.078	*k* = −9→9
1134 measured reflections	*l* = −5→6

### Bismuth pentafluoride Refinement

**Table:**

Refinement on *F*^2^	Primary atom site location: dual
Least-squares matrix: full	*w* = 1/[σ^2^(*F*_o_^2^) + (0.0237*P*)^2^ + 2.0256*P*] where *P* = (*F*_o_^2^ + 2*F*_c_^2^)/3
*R*[*F*^2^ > 2σ(*F*^2^)] = 0.016	(Δ/σ)_max_ < 0.001
*wR*(*F*^2^) = 0.042	Δρ_max_ = 0.90 e Å^−^^3^
*S* = 1.12	Δρ_min_ = −1.38 e Å^−^^3^
174 reflections	Extinction correction: SHELXL2018/3 (Sheldrick 2015b), Fc^*^=kFc[1+0.001xFc^2^λ^3^/sin(2θ)]^-1/4^
12 parameters	Extinction coefficient: 0.0080 (17)
0 restraints	

### Bismuth pentafluoride Special details

**Table:**

Geometry. All esds (except the esd in the dihedral angle between two l.s. planes) are estimated using the full covariance matrix. The cell esds are taken into account individually in the estimation of esds in distances, angles and torsion angles; correlations between esds in cell parameters are only used when they are defined by crystal symmetry. An approximate (isotropic) treatment of cell esds is used for estimating esds involving l.s. planes.

### Bismuth pentafluoride Fractional atomic coordinates and isotropic or equivalent isotropic displacement parameters (Å^2^)

**Table:**

	*x*	*y*	*z*	*U*_iso_*/*U*_eq_	
Bi	0.000000	0.000000	0.000000	0.0199 (2)	
F1	0.000000	0.000000	0.500000	0.0255 (15)	
F2	0.2859 (7)	0.0950 (7)	0.000000	0.0275 (8)	

### Bismuth pentafluoride Atomic displacement parameters (Å^2^)

**Table:**

	*U*^11^	*U*^22^	*U*^33^	*U*^12^	*U*^13^	*U*^23^
Bi	0.0225 (2)	0.0225 (2)	0.0147 (2)	0.000	0.000	0.000
F1	0.029 (2)	0.029 (2)	0.018 (3)	0.000	0.000	0.000
F2	0.0232 (17)	0.0320 (19)	0.0274 (19)	−0.0031 (14)	0.000	0.000

### Bismuth pentafluoride Geometric parameters (Å, º)

**Table:**

Bi—F2^i^	1.941 (4)	Bi—F2	1.941 (4)
Bi—F2^ii^	1.941 (4)	Bi—F1	2.1130 (5)
Bi—F2^iii^	1.941 (4)	Bi—F1^iv^	2.1130 (5)
			
F2^i^—Bi—F2^ii^	90.0	F2^iii^—Bi—F1	90.0
F2^i^—Bi—F2^iii^	90.0	F2—Bi—F1	90.0
F2^ii^—Bi—F2^iii^	180.0 (3)	F2^i^—Bi—F1^iv^	90.0
F2^i^—Bi—F2	180.0	F2^ii^—Bi—F1^iv^	90.0
F2^ii^—Bi—F2	90.0	F2^iii^—Bi—F1^iv^	90.0
F2^iii^—Bi—F2	90.0	F2—Bi—F1^iv^	90.0
F2^i^—Bi—F1	90.0	F1—Bi—F1^iv^	180.0
F2^ii^—Bi—F1	90.0	Bi^v^—F1—Bi	180.0

Symmetry codes: (i) −*x*, −*y*, −*z*; (ii) *y*, −*x*, −*z*; (iii) −*y*, *x*, *z*; (iv) *x*, *y*, *z*−1; (v) *x*, *y*, *z*+1.

### Fractional atomic coordinates and isotropic displacement parameters (Å^2^).

**Table:**

	*x*	*y*	*z*	*U_iso_*
Bi	0	0	0	0.0199 (2)
F1	0	0	1/2	0.0255 (15)
F2	0.2859 (7)	0.0950 (7)	0	0.0275 (8)
